# Anaemia and associated risk factors among pregnant women in Gilgel Gibe dam area, Southwest Ethiopia

**DOI:** 10.1186/1756-3305-5-296

**Published:** 2012-12-17

**Authors:** Million Getachew, Delenesaw Yewhalaw, Ketema Tafess, Yehenew Getachew, Ahmed Zeynudin

**Affiliations:** 1 Department of Biomedical Sciences, School of Health, Adama Science and Technology University, Asella, Ethiopia; 2 Departments of Biology, College of Natural Sciences, Jimma University, Jimma, Ethiopia; 3 Department of Information Communication Technology, College of Agriculture and Veterinary Medicine, Jimma University, Jimma, Ethiopia; 4 Department of Laboratory Technology and Pathology, College of Public Health and Medical Sciences, Jimma University, Jimma, Ethiopia

**Keywords:** Co- infection, Pregnant women, Soil Transmitted Helminths, Anaemia, Malaria, Ethiopia

## Abstract

**Background:**

Anaemia is known to be one of the outcomes of parasitic infection and it may result in impaired cognitive development, reduced physical work capacity and in severe cases increased risk of mortality, particularly during the prenatal period. The aim of this study was to determine the prevalence and associated risk factors of anaemia among pregnant women in Gilgel-Gibe dam area, southwestern Ethiopia.

**Methods:**

A cross-sectional community based study was conducted on 388 pregnant women living in three districts around Gilgel Gibe Dam area, southwestern Ethiopia. Socio-demographic and socio-economic data were collected from each participant. A single stool sample was also collected from each selected pregnant woman. Haemoglobin concentration was determined by the cyanmethemoglobin method. Plasmodium infection prevalence and intensity were assessed with thin and thick blood film examination.

**Results:**

Of the total 388 study participants, 209 (53.9%) were anaemic. Pregnant woman who were rural residents (Adjusted odds ratio (AOR) = 1.62, 95% C.I: 1.02-2.62, P= 0.042), not using insecticide treated nets (ITNs) during the study period (AOR = 2.84, 95% C.I: 1.33-6.05, p = 0.007), those who were Plasmodium malaria infected (AOR = 11.19, 95% C.I: 3.31-37.7, p= 0.01) and those with Soil Transmitted Helminth (STH) infections (AOR=1.82, 95% C.I: 1.16-2.87, p=0.001) had higher odds of being anaemic than those who were urban residents, using ITNs, free of Plasmodium malaria and Soil transmitted helminth infection, respectively. There was a significant correlation between increasing hookworm parasite load (r = −.110, *P*< 0.001), *Ascaris lumbricoides* (r = −.122, P < 0.001) and *Trichuris trichiura* (r = −.025, P < 0.001) and decreasing hematocrit values.

**Conclusion:**

The high prevalence of anaemia indicates it is currently a serious health problem of pregnant women living in Gilgel Gibe Dam area. Plasmodium malaria and soil transmitted helminth infections were significantly associated with anaemia. Antenatal care should promote de-worming and education on personal hygiene. Therefore, there is a need to design strategies that help to diagnose pregnant women for malaria and STH infections during their antenatal care (ANC) visit instead of testing for only haemoglobin (Hgb) levels and blood group.

## Background

Anaemia is a condition in which the number of red blood cells or their oxygen-carrying capacity is insufficient to meet physiologic needs, which varies by age, sex, altitude, smoking, and pregnancy status. Parasitic diseases, including helminth infections and *P. falciparum*, have long been recognized as important contributors to anaemia in endemic countries [1]. The effects of infection with a single helminth species on the risk of anaemia are also well documented, with risk correlated to infection intensity [2]. Hookworm causes iron deficiency anaemia through the process of intestinal blood loss and through nitric oxide (NO) release [3]. *Ascaris lumbricoides* and *Trichuris trichiuria* typically have little impact on iron status, and thus on anaemia. Because the mechanisms by which malaria and intestinal helminth infections cause anaemia differ, it is possible that their impact on anaemia are additive [4] and could exacerbate adverse birth outcomes [5].

Malaria due to *P. falciparum* also clearly contributes to anaemia throughout life and specifically during pregnancy. It is estimated that in sub-Saharan Africa 23 million pregnant women are exposed to malaria infection annually and approximately 400,000 pregnant women develop moderate or severe anaemia (haemoglobin < 80 g/L or hematocrit < 0.25) each year in sub-Saharan Africa as a result of malaria infection [6]. Soil transmitted helminths, especially hook worm infection is well known to cause anaemia. Data from the early 1990s suggested that 44 million of the developing world’s 124 million pregnant women harbored hookworm infection [7]; with 7.5 million in sub–Saharan Africa alone. Hookworm infection is considered a major health threat to adolescent girls and women of reproductive age, with adverse effects on the outcome of pregnancy [8]. In Ethiopia, anaemia prevalence of 41.9% and 51.9% was reported among pregnant women attending ANC from Jimma and Bushulo health centers*,* respectively [9,10].

Although several studies have been conducted in different parts of the world to understand the association between parasitic infections and anaemia among pregnant women, there is still not enough literature in Ethiopia. Therefore, this study was conducted to determine the current burden of parasitic infection and to assess the association between parasitic infection and anaemia among pregnant women living around Gilgel Gibe dam area, south west Ethiopia; so that the outcomes of the findings can help in the evidence- based decision to develop control intervention strategies to improve the health status of the most vulnerable groups i.e. pregnant women.

## Methods

### Study area, design and subjects

A cross-sectional community based study was conducted from August to September, 2011 in Gilge Gibe Dam area, 260 km south-west of Addis Ababa. The study area lies between latitudes 7°42’50”N and 07°53’50”N and between longitudes 37°11’22”E and 37°20’36”E, at an altitude of 1672–1864 m above sea level. The area has a sub-humid, warm to hot climate, receives between 1300 and 1800 mm of rain annually and has a mean annual temperature of 19°C. The rainfall pattern of the area is similar to other parts of Ethiopia with the long rainy season starting in June and extending up to September, while the short rainy season begins in March and extends to April/May. The main socio-economic activities of the local communities are mixed farming involving the cultivation of staple crops (maize, teff and sorghum), combined with cattle and small stock-raising. The study villages are located in Tiro-Afeta, Omo-Nada and Kersa districts (*weredas*). Houses are traditional type constructed of mud and wood, the majority with thatched roofs and very few with corrugated iron sheets.

**Figure 1:**
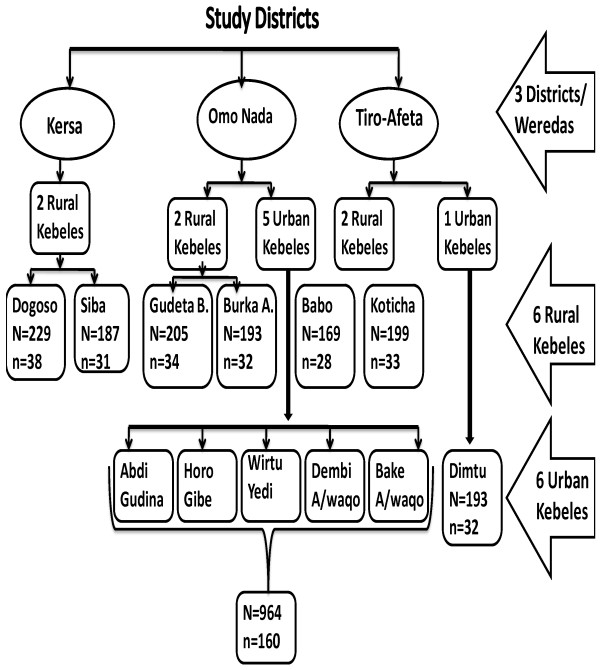
**Flow chart indicating the sampling procedure.** Where: N= the number of pregnant women in given rural Kebeles or urban Kebeles n= number of pregnant women sampled from rural Kebeles or urban Kebeles.

Multi-stage sampling was employed to select study households. Pregnant women were then re-selected from six rural and six urban *kebeles* using systematic sampling from the sampling frame, which was prepared after identifying the pregnant women and assessed through active detection using house- to- house visits (Figure 1). Sample size was determined using the formula for single population proportion by considering, 41.9 % proportion, prevalence of anaemia in pregnant women from Jimma, Ethiopia [10], 95% level of confidence, 5% margin of error and 5% none response rate. Thereby n = (1.96)2 0.419 (1–0.419)/ (0.05)2 =374, and adding 5% non response rate the final sample size was 393. Pregnant women were identified with the support of health extension workers (HEWs) who were stationed in each study Kebeles.

### Stool specimen collection and examination for intestinal parasites

After obtaining written consent from study participants, a single stool sample was collected from each participant. The study participants were provided with labeled screw capped stool containers and informed on how to collect about a 5g stool sample. The collected stool samples were immediately transported to Jimma University Clinical Laboratory where they were processed following the standard procedure using the McMaster concentration technique. The McMaster technique uses a counting chamber that enables a known volume of faecal suspension (2 × 0.15 ml) to be examined microscopically. Thus, if a known weight of faecal specimen and a known volume of flotation fluid were used to prepare the suspension, then the number of eggs per gram of faeces (epg.) was calculated [11]. Ten percent of the positive stool samples were randomly selected and cross- checked. The type of parasite and parasitic load were also recorded.

### Hematocrit determination

Capillary blood samples were collected from all the 388 pregnant women following aseptic technique. Haemoglobin concentration was determined by the cyanmethemoglobin method as described by Babara and Bates [12] and recommended by the International Committee for Standardization in Haematology (ICSH) [13] and WHO [14]. The haemoglobin cut of value for anaemia according to the WHO guidelines is 11g/dl, which is approximately equal to 33% hematocrit value [14]. Therefore, those pregnant women with HCT values less than 33% were categorized as anaemic women. Anaemic women were further categorized as women with mild anaemia, moderate anaemia and severe anaemia which corresponds to HCT values of ≥30 %< 33%, ≥21% <30% and <21%, respectively.

### Blood film preparation and examination of malaria parasites

The finger was cleansed with an alcohol-moistened swab, dried with a piece of dry cotton, and pricked with a disposable blood lancet. Through wiping off the first drop of blood, thick and thin films were made on the same slide. After being air-dried in a horizontal position, the slides were placed in a slide box and carefully transported to Asendabo Health Center, southwestern Ethiopia.

Before staining the blood films, the thin blood films were fixed in methanol for 30 sec. Then smears were stained with 10% Giemsa solution for 10 minutes. The staining techniques and blood film examination were conducted employing WHO guidelines [15]. Microscopic examination of thick films, using high power magnification for the presence of parasites and parasite species identification using thin films under a 100× oil immersion objective were carried out by an experienced laboratory technician and microscopist. In addition to the qualitative examination, parasitic load was determined following WHO guidelines [15].

### Socio-demographic and socio-economic survey

Data were collected by trained data collectors, who were well conversant,(speak, read and write) with the local language (*Afaan Oromo*). They were hired and trained on how to interview and adhere to the survey protocol. Each pregnant woman resident in the study village was assigned a household number, name identifying household member, age, and sex. The survey was conducted using a pre-tested, semi-structured questionnaire having both closed and open ended questions. The structured questionnaire was first developed in English and then translated into local language (*Afaan Oromo*) and administered in the local language to each pregnant woman. The questionnaire was developed to address the following categories: demographic characteristics, socio-economic factors, common human ailments in the area, STH and malaria related episodes and knowledge and perception questions related to STH and malaria transmission, causations, signs, symptoms, burden and severity of the disease, treatment seeking behavior, local prevention and control practices. A face- to- face interview schedule was arranged to collect relevant data from each pregnant women. Interviews were conducted privately to maintain confidentiality and avoid family and peer pressure.

### Data analysis

Data from both the laboratory and survey were checked and cleaned for completeness and consistency. Data were then analyzed using SPSS version 16.0 software package. For the analysis of demographic data descriptive statistics was employed. Point estimation of prevalence and intensity of STH and malaria infection and also odds ratios (OR) with a 95% confidence interval were computed to compare each of the two variables using chi-square test. Multivariable logistic regressions were also employed for those variables that had significant association with disease outcome to determine the main predictors of infection. P-value ≤ 0.05 was considered significant during the analysis

### Ethical consideration

Ethical clearance of the study was obtained from the Research Ethics Review Board of Jimma University. Permission from the community was sought before initiating the study by communicating the responsible zonal and district administrative offices through official letters from Jimma University. Similarly, community agreement and local oral consent was sought from village leaders through meetings with villagers. Individual informed oral and written consent were sought from each pregnant woman in the local language, *Afaan Oromo*, for all literate pregnant women. An independent literate witness from village leaders confirmed verbal consent for illiterate pregnant woman after the objectives and the nature of the study were explained to the participants so as to get their oral and written consent to be involved in the study voluntarily. Data collected during the survey from each study participant and results of laboratory tests were kept confidential. Results of participants with parasitic infections, malaria and/or intestinal helminth, and low HCT level were sent, as soon as possible, to nearby health facilities for treatment and medical consultation in the ANC. Those pregnant women found infected were referred for treatment.

## Results

Out of a total sample size (393), 388 (98.7%) pregnant women responded and there was a non response rate of 0.76%. Of the total 388 study participants, 209 (53.9%) were anaemic. The minimum, maximum and mean HCT values were 18%, 48% and 32.7%, respectively. Of those 209 anaemic women; 115 (55%), 88 (42.1%), and 6 (2.9%) had mild, moderate and severe anaemia, respectively (data not shown). There was a significant difference in prevalence of anaemia between place of residence (X^2^ = 11.19, P = 0.001), use of human faeces as a fertilizer (X^2^=8.26, P= 0.004), ITNs utilization (X^2^= 4.67, p= 0.031), habit of walking barefoot (X^2^ = 5.95, p = 0.01) occupation (X^2^ = 19.10, p = 0.001) and water sources (X^2^ = 13.13, P = 0.01) (Table 1).

**Table 1:** Association of anaemia with socio-demographic characteristics and environment related factors among pregnant women in Gilgel Gibe hydropower dam area, southwest Ethiopia, August to September 2011

**Variable**		** n**	**Anaemia**		** Χ**^**2**^	**P-value**
			** Anaemic**	**Non Anaemic**		
			**N=209 (%)**	** N=179 (%)**		
**Place of residence**	Urban	192	87 (45.3%)	105 (54.7%)	11.19	0.001*
	Rural	196	122 (62.2%)	74 (37.8%)		
**Age group**	16-20	128	69 (53.9%)	59 (46.1%)	5.55	0.235
	21-25	115	62 (53.9%)	53 (46.1%)		
	26-30	110	54 (49.1%)	56 (50.9%)		
	31-35	27	17 (63%)	10 (37%)		
	36-40	8	7 (87.5%)	1 (12.5%)		
**Occupation**	Housewife	191	86 (45%)	105 (55%)	19.10	0.001*
	Farmer	176	114 (45.3%)	62 (54.7%)		
	Daily labourer	4	0 (0%)	4 (100%)		
	Civil servant	13	7 (53.8%)	6 (46.2%)		
	Business man	4	2 (50%)	2 (50%)		
**Parity**	Primigravida	125	66 (52.8%)	59 (47.2%)	0.08	0.772
	multigravida	263	143 (54.4%)	120 (45.6%)		
	Third trimester	143	90 (63%)	53 (37%)		
**Water source**	Pipe	171	76 (44.5%)	95 (55.5%)	13.13	0.01*
	Protected well	74	41(55.4%)	33 (44.6%)		
	Unprotected Well	4	3 (75%)	1 (25%)		
	River/pond	53	32 (60.4%)	21 (39.6%)		
	Spring	86	57 (66.3%)	29 (33.7%)		
**Human faeces as fertilizer**	Yes	32	25 (78.1%)	7 (21.9%)	8.26	0.004*
	No	356	184 (51.7%)	172 (48.3%)		
**ITN utilization**	Yes	348	181 (52%)	167 (48%)	4.67	0.031*
	No	40	28 (70%)	12 (30%)		
**Habit of walking bare foot**	Yes	273	158 (57.9%)	115 (40.1%)	5.95	0.010*
	No	115	51 (44.3%)	64 (55.7%)		

* Significant at (p < 0.05).

Socio demographic and parasitic infections were analyzed in relation to anaemia by bivariate and multivariate logistic regression analyses model. In the bivariate logistic regression analysis, anaemia was associated significantly with residence, use of human faeces as fertilizers, use of ITNs to prevent mosquito, habit of working bare foot, Plasmodium and STH infection (Table 2).

**Table 2:** Parameter estimates from univariate Logistic regression model predicting the likelihood that a pregnant woman is anaemic, Gilgel Gibe Dam area, southwest Ethiopia, August to September 2011

**Variable**	**n**	**Anaemic**	**OR**	**95%CI**	**P-value**
**Place of residence**					
Rural	196	87(45.3)	1.99	1.33 - 2.98	0.001*
Urban	192	122(62.2)	1		
**Family income**					
<500 birr	108	55(50.9)	0.98	0.45 - 2.13	0.704
500-1000	247	137(55.5)	1.17	0.56 - 2.43	
>1000	33	17(51.50	1		
**Number of children**					
More than one child	210	113(53.9)	1.04	0.61-2.22	0.897
One child	63	30(56.6)	1.16	0.67-1.62	
No children	125	66(52.8)	1		
**Parity**					
multigravida	125	66(52.8)	1.06	0.69-1.63	0.772
primegravida	263	143(54.4)	1		
**Trimester**					
3^rd^	143	90(62.9)	1.17	0.68-2.02	0.560
2^nd^	157	67(42.7)	0.52	0.30-0.87	
1^st^	88	52(59.1)	1		
**Use of human faeces as fertilizer**					
Yes	32	25(78.1)	3.34	1.41-7.92	0.006*
No	356	184(51.7)	1		
**Use of ITN to prevent mosquito bites**					
No	40	28(70.0)	2.15	1.06-4.37	0.034*
Yes	348	181(52.0)	1		
**Habit of working bare foot**					
Always	26	19(73.10)	3.41	1.33-8.73	0.015*
Some times	247	139(56.3)	1.61	1.03-2.52	
Not at all	115	51(44.3)	1		
**Malaria**					
Positive	45	42(93.3)	14.75	4.48-48.51	0.001*
Negative	343	167(48.7)	1		
**STH infection**					
Positive	159	105(66.0)	2.34	1.54-3.55	0.001*
No parasite	229	104(45.5)	1		

*significant at p < 0.05, OR- odds ratio, C.I- confidence interval.

Pregnant women who were rural residents (AOR = 1.62, 95% C.I: 1.02-2.62, P= 0.042), not using ITN during the study period (OR = 2.84, 95% C.I: 1.33-6.05, p = 0.007), those who were Plasmodium infected (OR = 11.19, 95% C.I: 3.31-37.7, p= 0.01) and those who had STH infection (OR=1.82, 95% C.I: 1.16-2.87, p=0.001) were found to be anaemic compared to those who were urban residents, using ITN, free of Plasmodium and Soil transmitted helminth infection, respectively (Table 3).

**Table 3:** Parameter estimates from multivariate logistic regression model predicting the likelihood that a pregnant woman is anaemic, Gilgel Gibe dam area, southwestern Ethiopia, 2011

**Variable**	**n**	**No (%) p anaemic**	**AOR**	**95% CI**	**p-value**
**Place of residence**					
Rural	196	87 (45.3)	1.63	1.02-2.62	0.042*
Urban	192	122 (62.2)			
**Use of human faeces as fertilizer**					
Yes	32	25 (78.1)	1.58	0.54-4.65	0.40
No	356	184 (51.7)			
**Use of ITN to prevent mosquito bite**					
No	40	28 (70.0)	2.84	1.33-6.05	0.007*
Yes	348	181 (52.0)	1		
**Habit of walking bare foot**					
Always	26	19 (73.1)	1.92	0.63-5.89	0.41
Some times	247	139 (56.3)	1.29	0.76-2.16	
Not at all	115	51 (44.3)	1		
**Malaria**					
Positive	45	42 (93.3)	11.19	3.31-37.7	0.01*
Negative	343	167 (48.7)	1		
**STH infection**					
Positive	159	105 (66.0)	1.82	1.16-2.87	0.001*
No parasite	229	104 (45.5)	1		

* significant at p < 0.05, AOR = Adjusted Odds Ratio, C.I = Confidence Interval.

Assessment was also carried out to determine the effect of hookworm, malaria and the whole STH parasite load, and infection on the severity of anaemia (Table 4). The study findings indicated that hookworm infection (P = 0.002), malaria (p < 0.001) and hookworm/Plasmodium infection (p < 0.001) showed significant association with anaemia. There was a significant correlation between increasing hookworm parasite load (r = −0.110, *P* < 0.001), *A. lumbricoids* (r = −0.122, P < 0.001) and *T. trichuira* (r = −0.025, P < 0.001) and decreasing hematocrit values. The rate of severe anaemia levels in hookworm loads greater than 1,000 ova/gram of stool was higher (data not shown).

**Table 4:** Association of STHs and malaria with anaemia among pregnant women in Gilgel Gibe Dam area, 2011

**Variable**		**n**	** Anaemic**	** Χ**^**2**^	**P-value**
**STH infection**	*T. trichiura*	13	11 (84.6%)	5.12	0.021
	*A. lumbricoides*	58	32 (55.2%)	0.05	0.472
	Hookworm	114	78 (68.4%)	13.76	0.002
**STH and malaria infection**	Only STH infection	129	78 (60.5%)	3.39	0.041
	Only malaria infection	15	15 (100%)	13.36	<0.001
	Malaria/STH infection	30	27 (90%)	17.08	<0.001

## Discussion

Anaemia is one of the most common outcomes of malaria and STH infection. The prevalence of anaemia was 53.9%; with minimum, maximum and mean Hematocrit values of 18%, 48% and 32.7%, respectively. This result was almost similar to that of anaemia prevalence in Bushulo health centers, Ethiopia 51.9% [10] but slightly higher than the anaemia prevalence previously reported from Jimma health center 41.9% [9], Asendabo health center 23% [16], and Peru 47.31% [17]. Our findings are close to the figures reported by UNO [18] and a small variation from other studies may be due to the selection of study population.

From the total of 209 anaemic women; 115 (55%), 88(42.1%), 6 (2.9%) were with mild, moderate and severe anaemia, respectively. This finding is higher than the study conducted on pregnant women in Malaysia, which reported 45%, 9.8%, and 1.85% with mild, moderate and severe anaemia, respectively [19].

Our findings indicated that pregnant women from rural residences, not using ITN during the study period, those who were Plasmodium infected and those with STH infection were highly likely to be anaemic compared to those from urban residences, using ITN, free of Plasmodium and STH infection.

In the current study those pregnant women who had a habit of walking bare foot had high anaemia prevalence (57.8%). Walking barefoot may predispose to hookworm infection and consequetly may result in iron deficiency anaemia especially in pregnant women [10]. Hookworm infection rate was also associated with anaemia in which those pregnant women infected with hookworm have a 2.4 times higher risk of developing anaemia, as 68.4% of the pregnant women infected with hookworm were anaemic. This finding is similar to the findings of other similar studies [10,20]. There was a significant correlation between increasing hookworm parasite load, *A.lumbricoides* and *T. trichiura* and decreasing hematocrit values. This shows that as the helminth parasitic load increased the hematocrit level decreased; as a result the risk of developing anaemia increased. This result was comparable to a study from Peru, where there was a significant correlation between increasing hookworm egg counts and decreasing haemoglobin levels [17]. In this study prevalence of anaemia in pregnant women was higher in malaria and STH infected pregnant women, which is in agreement with the findings of many studies [5,10,20,21].

There are, of course, limitations in this study. Even though this study tried to address some important factors, other factors, such as other nutritional deficiencies (including folate, vitamin B12 and vitamin A), acute and chronic inflammation, and inherited or acquired disorders that affect haemoglobin synthesis, red blood cell production or red blood cell survival, which can all cause anaemia, were not addressed. In this study anaemia in pregnancy was also not standardized for residential elevation above sea level (altitude). In addition, the study was not out of the limitations of cross sectional study like identifying the cause and effect relationship.

## Conclusion

Anaemia, STH infection and malaria are currently serious health problems of pregnant women living in Gilgel Gibe Dam area. Rural residence, ITN utilization, and Plasmodium and STH infection were found to be significantly associated with anaemia in the study area. The severity of anaemia is pronounced more when pregnant women infected with STH are co-infected with malaria. The high prevalence of anaemia indicates it is currently a serious health problem of pregnant women living in Gilgel Gibe Dam area. Antenatal care should promote de-worming and education on personal hygiene. Therefore, there is a need to design strategies that would help to diagnose pregnant women for malaria and STH infection during their antenatal care (ANC) visit instead of testing only for haemoglobin (Hgb) level and blood group.

## Competing interests

The authors declare that they have no competing interests.

## Authors’ contributions

MG conceived the study, designed, participated in data collection, conducted data analysis, drafted and finalized the manuscript for publication. DY, YG, AZ and KT assisted in data collection and reviewed the initial and final drafts of the manuscript. MG, DY, YG, AZ and KT interpreted the results, and reviewed the initial and final drafts of the manuscript. All authors read and approved the final manuscript.
